# ‘People like me don’t do well at school’: The roles of identity compatibility and school context in explaining the socioeconomic attainment gap

**DOI:** 10.1111/bjep.12494

**Published:** 2022-03-01

**Authors:** Matthew J. Easterbrook, Marlon Nieuwenhuis, Kerry J. Fox, Peter R. Harris, Robin Banerjee

**Affiliations:** ^1^ 1948 School of Psychology University of Sussex UK; ^2^ Faculty of Behavioural, Management, and Social Science University of Twente The Netherlands; ^3^ School of Applied Social Science University of Brighton UK

**Keywords:** attainment gaps, education, identity compatibility, school context, socioeconomic status

## Abstract

**Background:**

School students who are eligible for reduced or free school meals (FSM) – an indicator of economic disadvantage – have lower academic attainment than their peers.

**Aims:**

We investigated whether identity compatibility – the perceived compatibility between one’s social identities and the stereotype of a high‐achieving student – contributes to this socioeconomic attainment gap, and whether the association between socioeconomic status and identity compatibility is moderated by school context.

**Sample:**

Our sample was 4,629 students aged 15–16 years old across 29 schools in England.

**Method:**

We assessed students’ perceptions of identity compatibility via self‐report questionnaires 8 months prior to them taking national, standardized exams.

**Results:**

Multilevel regression analyses revealed a negative indirect effect from eligibility for FSM to exam results via identity compatibility. These effects existed even while accounting for students’ gender and language status, other psychological variables known to predict academic attainment, and their previous exam results. Furthermore, school context moderated the relationship between FSM eligibility and identity compatibility. In line with the identities in context model of educational inequalities, there was a significant negative association between FSM and identity compatibility only for students attending schools in which there was previously a relatively large socioeconomic attainment gap.

**Conclusions:**

Our results demonstrate the importance of social psychological variables in explaining educational inequalities, and of the local educational context in determining the educational experience of students from lower socioeconomic status backgrounds.

## Background

In many Western countries, students from families of lower socioeconomic status attain lower grades in national exams, have lower progression rates to higher education, and consequently have less economically prosperous life trajectories than their wealthier peers (Department for Education, 2018, 2020; Education Endowment Foundation, 2018; Nation's Report Card, 2019). In England, the socioeconomic attainment gap is evident at age 5 and increases as students progress through the compulsory education system. When students are 16 years old, it has been estimated that it would take two and a half years of additional schooling to bring the academic performance of economically disadvantaged students up to the same level as their wealthier peers (a gap that is equivalent to 0.66 standard deviations; Easterbrook & Hadden, 2021).

These inequalities are not explained solely by differences in academic ability (Jerrim, Chmielewski, & Parker, 2015; Machin & Vignoles, 2005; Nieuwenhuis, Manstead, & Easterbrook, 2019) or by the structural and economic inequalities that exist between these groups (Easterbrook & Hadden, 2021). Indeed, meta‐analyses have suggested that a substantial proportion of the variation in attainment between groups can be attributed to *social psychological differences* between them (Walton & Spencer, 2009).

In this article, we investigate the role of identity compatibility – the perception that one’s social identities or background are compatible with the stereotypes associated with academic achievement (Jetten, Iyer, Tsivrikos, & Young, 2008) – in explaining socioeconomic inequalities in results from national exams taken at age 16 in England. We draw on the identities in context model of educational inequalities (Easterbrook & Hadden, 2021; Easterbrook, Hadden, & Nieuwenhuis, 2019) to predict that identity compatibility will help to explain the socioeconomic attainment gap, but also that the association between lower socioeconomic status (SES) and identity compatibility will vary across schools in such a way that it will be more strongly negative in schools with larger pre‐existing socioeconomic attainment gaps. We elaborate on these predictions and the background research that led to them, below.

### Identity incompatibility

Within education, identity compatibility refers to the perception of compatibility or conflict between one’s social identities or social background and the stereotype of a successful student. Given the large existing socioeconomic attainment gap in the UK and the overrepresentation of individuals from higher socioeconomic status (SES) backgrounds in positions associated with educational attainment and prestige (Arulampalam, Naylor, & Smith, 2005; Jones, 2016; Reay, 2017), the stereotypes of academic achievers and those of individuals with high SES are likely to heavily overlap and thus be perceived as highly compatible (Easterbrook et al., 2019). In contrast, students from groups that have been historically marginalized in education – such as students from lower socioeconomic groups or from certain ethnic minorities – are likely to have few, if any, role models in positions associated with educational achievement and thus may perceive educational success as incompatible and at odds with their group’s social identity.

Identity compatibility has been shown to be associated negatively with feelings of isolation and alienation within educational institutions, and positively with academic aspirations and performance (Dasgupta, 2011; Oyserman, Bybee, & Terry, 2006; Oyserman, Johnson, & James, 2011; Sheldon & Schüler, 2011). We therefore expect that lower‐socioeconomic students will perceive that their social identities are *in*compatible with the stereotype of high academic achievers, and that this will contribute to the socioeconomic attainment gap in English schools (Easterbrook & Hadden, 2021).

Research focusing on gender has shown the importance of identity compatibility in explaining gender inequalities in STEM subjects. Reflecting women’s historical underrepresentation in STEM fields and the masculine stereotypes associated with STEM (Eagly & Koenig, 2021), women tend to perceive lower levels of compatibility between their gender identity and the stereotypes about successful STEM students than men do. Lower levels of identity compatibility in STEM are associated with reduced belonging, confidence, motivation, and performance in STEM fields, and thus contribute to gender‐based inequalities in STEM outcomes (Ahlqvist, London, & Rosenthal, 2013; Good, Rattan, & Dweck, 2012; Rosenthal, London, Levy, & Lobel, 2011; Settles, 2004; Settles, Jellison, & Pratt‐Hyatt, 2009).

There is evidence that identity compatibility is also associated strongly with SES, although this body of research focuses exclusively on Higher Education. University students from lower (vs. higher) SES backgrounds have been found to perceive less identity compatibility between their backgrounds and the stereotype of a university student, and this in turn predicts lower levels of identification with the university (Jetten et al., 2008), less positive affect, greater levels of depressive symptoms over time (Iyer, Jetten, Tsivrikos, Postmes, & Haslam, 2009), less social integration, and poorer academic performance (Veldman, Meeussen, & van Laar, 2019). Other evidence has shown that, even while controlling for academic grades, students in the UK from lower (vs. higher) socioeconomic positions were more likely to plan to apply to lower ranking universities, and that this association was partly explained by a lower sense of compatibility between their social background and being a university student (Nieuwenhuis et al., 2019). Identity compatibility therefore helps to explain SES inequalities in educational outcomes among university students.

In this article, we extend the concept of identity compatibility to refer to the perceived fit between one’s socioeconomic background and the stereotype of someone who does well *at school*. We argue that the social identities of those from lower SES backgrounds are unlikely to incorporate academic success or academic possible selves (Oyserman et al., 2006), that the stereotypically high‐achieving student is considered to be of higher SES, and thus that lower SES students will perceive less compatibility between their social backgrounds and the stereotypes of high achieving students.

Students from lower SES backgrounds have on average lower performance in national exams, lower rates of progression to higher education (Department for Education, 2020), and are underrepresented in positions associated with educational attainment (Arulampalam et al., 2005; Jones, 2016). These sociocultural factors contribute to the stereotype that high academic achievers are from high SES backgrounds (Eagly & Koenig, 2021), and feed into those groups’ social identities (Easterbrook et al., 2019; Manstead, Easterbrook, & Kuppens, 2020). Indeed, there is evidence that SES is associated strongly and positively with stereotypes of competence (Cuddy, Fiske, & Glick, 2007; Fiske, 2010), and that there are descriptive and prescriptive stereotypes that link lower SES with academic incompetence among adults and children (Batruch, Autin, & Butera, 2017; Croizet & Claire, 1998; Goudeau & Croizet, 2016). Those of lower SES also experience discrimination, stigma, and threat within educational institutions (Easterbrook et al., 2019; Hadden, Easterbrook, Nieuwenhuis, Fox, & Dolan, 2020; Reay, 2017), which tend to be associated with belonging to a group that is expected (by others and the self) to perform poorly (Steele & Aronson, 1995). It is likely, therefore, that low SES students will be stereotyped as academically less competent, and that current or potential educational success will not be a meaningful part of the social identity of students from low SES backgrounds (Oyserman et al., 2006).

To our knowledge, the only study relevant to the investigation of identity compatibility among a sample of school students is an intervention study conducted in the United States examining academic possible selves (Oyserman et al., 2006). Research into academic possible selves has demonstrated that students from lower (vs. higher) SES families have fewer academic possible selves and fewer behavioural strategies to achieve those that they do possess (Oyserman et al., 2011). The intervention sought (among other things) to encourage low‐income US middle‐school students to see their social identities as compatible with the possible future identity of someone who successfully completed the academic year (Oyserman et al., 2006). This was found to improve students’ academic performance (Grade Point Average), suggesting that compatibility between one’s social identities and the stereotype of a successful student is positively related to academic outcomes among school students. Building on the above research, we expect that identity compatibility will be lower among students from lower socioeconomic backgrounds, and that this will be negatively associated with their academic performance.

### The school context

The identities in context model of educational inequalities (Easterbrook & Hadden, 2021; Easterbrook et al., 2019) details various sociocultural factors that, if present in the local context and relevant to a particular group, are likely to contribute to a sense of threat or the perception of identity *in*compatibility among members of that group. The sociocultural factors outlined by the model are negative stereotypes about the group’s performance, their historical performance, the presence and availability of role models, the group’s numerical representation in positions and institutions associated with educational attainment, and their orientation towards education, including cultural capital, norms, and values. If one or more of these factors is present for a particular group, then that group is likely to experience a sense of threat and perceive identity *in*compatibility, and thus social psychological factors are likely to dampen that group’s educational success and ambitions.

Here, we focus on one of these sociocultural factors – the group’s historical performance. We empirically test predictions derived from the identities in context model which suggest that historical performance of SES groups will moderate the association between students’ socioeconomic background and their identity compatibility (Easterbrook & Hadden, 2021). Sociocultural variables such as a group’s relative performance can signal the value or status of that group within the relevant context, and so contribute to the content and meaning of their social identities (Manstead et al., 2020; Uskul & Oishi, 2020). Within a school, a large socioeconomic attainment gap is likely to act as a signal indicating that students from lower socioeconomic groups are not expected to do well in education and that educational success is something that members of low SES groups are unlikely to achieve. This may mean that members of low SES groups develop few, if any, academic possible selves, and will fuel perceptions of identity *in*compatibility among low SES students (Easterbrook et al., 2019; Oyserman et al., 2006, 2011). According to this theorizing, the socioeconomic attainment gap alters the meaning of groups’ social identities and so should only moderate the association between group membership and identity compatibility. We do not expect it to moderate the direct association between group membership and attainment, nor the association between identity compatibility and attainment, which we expect to be stable across contexts.

Although our prediction that the school context will moderate the association of socioeconomic position with identity compatibility has never been directly investigated, some results suggest it has merit. Firstly, there is evidence that promoting the visibility of high‐performing members of poorly performing groups can boost the belonging, compatibility, and performance of members of those groups (Andriessen, Phalet, & Lens, 2006; Hernandez, Rana, Rao, & Usselman, 2017; Oyserman et al., 2006; Stout, Dasgupta, Hunsinger, & McManus, 2011). This implies that group members do indeed perceive and react to visible indicators of their group’s academic performance.

Secondly, two papers suggest that the size of attainment gaps can moderate the association between group membership and social psychological variables related to social identities. A meta‐analysis found that the average effect size of interventions designed to reduce the effects of stereotype threat on women’s maths performance is smaller in contexts that have smaller gender‐based attainment gaps in mathematics (Picho, Rodriguez, & Finnie, 2013). And an intervention study found that self‐affirmation – an intervention designed to reduce the negative effects of stereotype threat on attainment – was most effective at raising the attainment of African American and Latino students in schools that had larger pre‐existing ethnic attainment gaps and a smaller proportion of ethnic minority students (Borman, Grigg, Rozek, Hanselman, & Dewey, 2018). These results presumably arise because, in contexts with smaller attainment gaps, the lower status group members are less threatened and perceived their social identities as more compatible with academic success. This leaves less scope for social psychological interventions that target social identity processes to improve academic performance (see also Easterbrook & Hadden, 2021; Manstead et al., 2020).

No studies have investigated such moderation among SES groups. Yet, the socioeconomic attainment gap is heterogeneous across regions and schools (Department for Education, 2020) and is, we argue, likely to feed into the stereotypes and social identities of students from lower‐ and higher‐socioeconomic groups in the local context, fuelling their perceptions of identity compatibility. We expect, therefore, that, in schools with larger socioeconomic attainment gaps, low SES students will have lower levels of identity compatibility and, in turn, poorer academic performance. In contrast, in schools with small or non‐existent socioeconomic attainment gaps, low SES students will have similar levels of identity compatibility to higher SES students, with correspondingly smaller differences in attainment.

### The current study

We report results from a study conducted in 29 English secondary schools with 15–16‐year‐old school students. Students completed self‐report questionnaires that contained measures of identity compatibility in September, at the beginning of the academic year. These were later matched with students’ demographic characteristics and their results from national standardized exams (General Certificate of Secondary Education exams) taken in May, at the end of the same academic year. We also collated Government data on the socioeconomic attainment gap in the previous year for all schools and used this in our multilevel models to directly test our hypothesis that the pre‐existing, school‐level, socioeconomic attainment gap will moderate the association between SES – which we measure using the proxy variable eligibility for free school meals (FSM) – and identity compatibility. Our prediction is specific to the FSM‐identity compatibility association, although, for completeness, we also investigate whether the school‐level socioeconomic attainment gap moderates the FSM to exam results association, and the identity compatibility to exam results association. We expect that it will not moderate these associations.

We aim to test the unique role of identity compatibility in explaining the socioeconomic attainment gap, and of the FSM attainment gap variable in moderating the FSM‐identity compatibility slope. To test the specificity of these predictions, we also include in our models two other psychological variables that have been found to robustly and strongly predict academic attainment, namely academic self‐concept and academic effort (Marsh et al., 2006). This allows us to examine the unique and additional predictive utility of identity compatibility while accounting for these well‐validated variables. We expect that identity compatibility will mediate the association between FSM and exam results, whereas academic self‐concept and academic effort will not do so. In addition, we expect that the association of FSM with identity compatibility will be moderated by the school‐level socioeconomic attainment gap, whereas the associations of FSM with academic self‐concept and academic effort will not be moderated by this variable.

We thus test whether academic self‐concept and academic effort mediate the association between FSM and exam results, expecting that they will not, and whether the associations of FSM with academic self‐concept and self‐reported effort are moderated by the school‐level socioeconomic attainment gap, again expecting that they will not be. We also include gender and whether English was an additional language (EAL) – which are often associated with attainment (Department for Education, 2020) – in our models, and explore whether the psychological variables mediate any attainment gaps between these groups.

The study was part of a larger randomized control trial evaluating the impact of a psychological intervention. The survey was conducted before any aspect of the intervention was implemented, however, and so was not confounded by the condition that students were allocated to.

**Figure 1:**
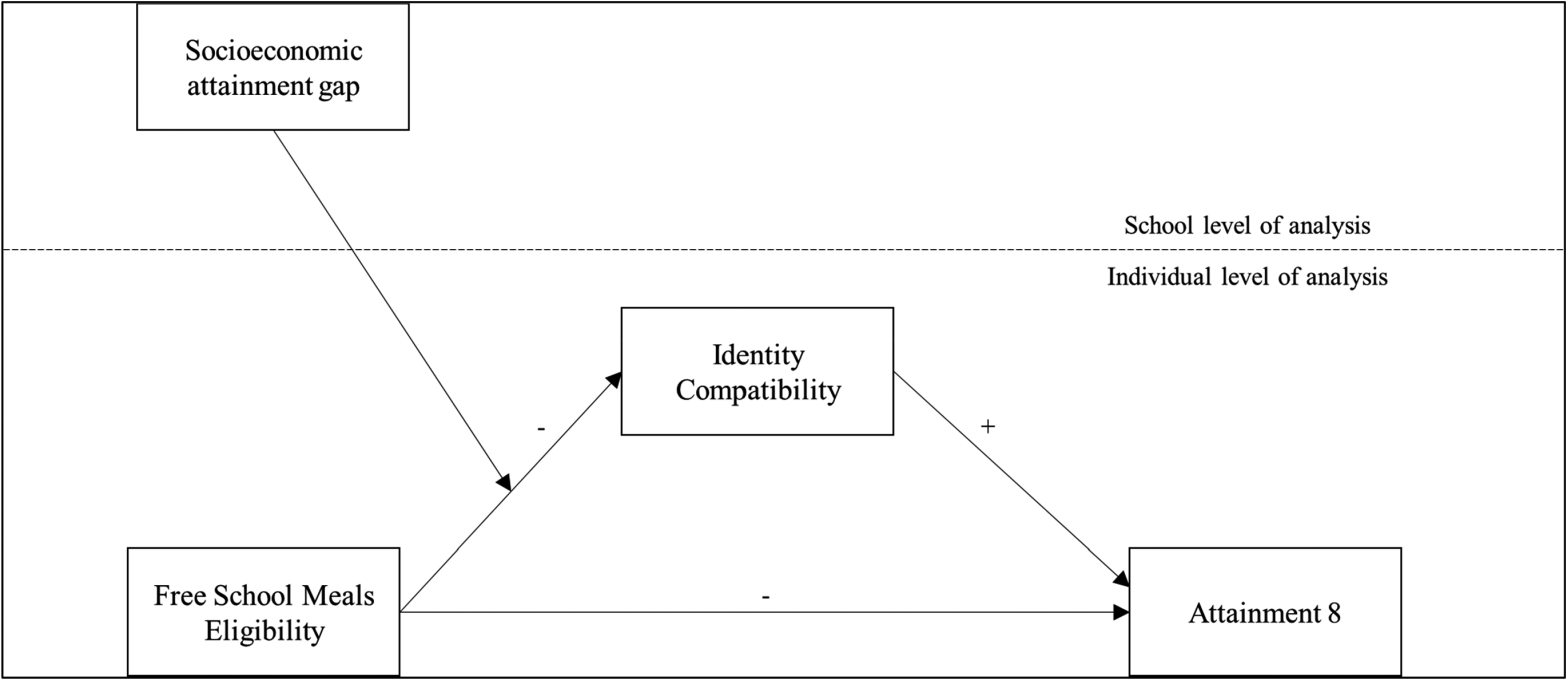
Diagram showing the theoretical predictions.

We test the following hypotheses, which are also represented in Figure 1:

Hypothesis 1 Eligibility for FSM will be associated negatively with students’ exam grades.

Hypothesis 2 Identity compatibility will be associated positively with students’ exam grades.

Hypothesis 3 Eligibility for FSM will be associated negatively with identity compatibility.

Hypothesis 4 There will be a significant negative indirect effect from FSM eligibility to exam grades via identity compatibility.

Hypothesis 5 The relationship at the individual‐level of analysis between FSM eligibility and identity compatibility will be moderated by the school‐level variable socioeconomic attainment gap (i.e., a cross‐level interaction), such that the association will be more strongly negative in schools with larger socioeconomic attainment gaps.

## Method

### Procedure

Twenty‐nine schools participated in this study.^1^ We planned for all Year 11 (age 15–16) students in the 29 participating schools to complete a survey in personal tutor time in the first 2 weeks of the academic year (September 2016). In the event, 27 schools returned the completed surveys. Most schools were recruited through brief recruitment presentations given at conferences for head‐teachers, whereas a minority were recruited through the researchers’ existing contacts. If head‐teachers expressed an interest in taking part, we sent them a memorandum of understanding and a consent form, which they were required to sign in order for their school to take part in the study. We sent information sheets and opt‐out consent forms to the parents/guardians of all students in Year 11, allowing at least 2 weeks for them to be returned (< 0.1% of students opted‐out). Schools were given a £1,000 honorarium for taking part. We recruited 30 schools, although one school dropped out before the study began, leaving 29. All Year 11 students in the schools took part unless they opted out or were absent from their tutorial time for 4 weeks. The study received ethical approval from all the relevant institutional ethics boards and adhered to all relevant ethical guidelines.

### Participants

The final sample that was analysed was composed of 4,629 Year 11 students, aged 15–16‐years old, from 29 English secondary schools. Of those, 26.9% were eligible for FSM within the last 6 years, the same as the national average (27%), 51.4% were females (national average: 46%), and 8.1% had English as an Additional Language (EAL, national average: 19%). More information about the initial sample, the analysed sample, the sample who completed the survey, and full descriptive statistics for the analysed sample (Table S1) are provided in the Supporting Information. The year groups ranged in size across the 29 schools, from *n* = 102 to *n* = 314. The proportion of pupils eligible for FSM also varied across schools, from 10% to 55%. One school was a girls‐only school, and all the others were mixed genders. All were state rather than private schools, three were Christian schools, three were secondary modern schools, and the rest were comprehensive schools.

### Measures

The following measures that are directly relevant to our hypotheses were included in a larger questionnaire relevant to the intervention. See Table S3 in the Supporting Information for a correlation matrix for all variables.

#### Student characteristics and academic attainment

Schools provided us with Unique Student Numbers (UPN) for all participating students, as well as whether students had English as an Additional Language (EAL, 0 = no, 1 = yes). We used the UPNs to retrieve data from the National Pupil Database (NPD, a government controlled centralized database) including whether students were eligible for FSM at any point in the last 6 years (our key measure of socioeconomic status, 0 = no, 1 = yes), gender (male = 0, female = 1), and attainment data.

Our primary outcome variable was students’ attainment 8 scores, which are the average grade across eight subjects in their General Certificate of Secondary Education (GCSE) assessments (taken at the end of Year 11), with double weighting for English and mathematics (Department for Education, 2016). We had attainment 8 data for *n = *5,079 students (*M* = 44.40, *SD* = 18.51, *range* = 0–87.50; higher values indicate superior performance). We controlled for students’ prior grades by including the mean of their total score in the Standardized Attainment Tests (SATs) for Key Stage 2 (KS2) English and for KS2 Mathematics (the only two subjects in which all students sit these exams), taken at age 10–11, labelled KS2 score. We had KS2 mathematics scores for *n = *5,046 students (*M* = 67.36, *SD* = 20.17, *range* = 5–10) and KS2 English scores for *n = *4,999 students (*M* = 71.49, *SD* = 14.46, *range* = 23–100; KS2 score, *M* = 69.14, *SD* = 16.22, *range* = 5–99.50).

#### Identity compatibility

We adapted measures of identity compatibility used in previous research (Iyer et al., 2009; Nieuwenhuis et al., 2019) to create a three‐item measure of identity compatibility. We provided the following definition of social background before the items: ‘The next questions are about you and your social background. By social background, we mean people who are from the same social class or community as you, who live in the same types of places as you, and who do similar things as you, and whose family has similar amounts of money and do similar sorts of things as yours.’ The items were ‘Working hard at school fits with my social background’, ‘My background is compatible with someone who does well in school’, and ‘People with my social background usually get good grades at school’. Responses were made on a 7‐point response scale with the anchors *Strongly disagree (1), Neither agree nor disagree (4),* and *Strongly agree (7)*. We took the mean of the three items as our measure of identity compatibility. The scale had good internal reliability (McDonald’s Ω = .853, *p* < .001).

#### Academic effort and academic self‐concept

We measured academic effort using the mean of slightly adapted versions of the four items from the Student Approaches to Learning questionnaire, and measured academic self‐concept using the mean of the three items from the same questionnaire (Marsh et al., 2006). An example academic effort item is ‘I work as hard as possible’. An example academic self‐concept item is ‘I'm good at most school subjects’. Responses were given on a 7‐point scale from *Strongly disagree* to *Strongly agree*. Both academic effort (McDonald’s Ω = .880, *p* < .001) and academic self‐concept (McDonald’s Ω = .834, *p* < .001) had good internal reliability.

#### Socioeconomic attainment gap

To test our hypothesis regarding school context, we collated data from the UK government’s website (https://www.compare‐school‐performance.service.gov.uk/) regarding the percentage of disadvantaged (FSM) and non‐disadvantaged (not FSM) 15–16‐year‐old students in each school who were awarded A* to C grades inclusive for English, Mathematics, and at least three other subjects in the GCSE exams taken in May and June 2016 (the year before our survey and attainment measures). The percentage of students at a school achieving A* to C grades is typically used by the UK Government as its criterion for good academic attainment. We computed the gap between disadvantaged and non‐disadvantaged students for each school by subtracting the percentage of disadvantaged students who achieved A* to C grades from the percentage of non‐disadvantaged students who achieved these grades. This is, therefore, a school‐level variable that we include in our models below as a school‐level predictor and in cross‐level interactions (*M* = 0.27, *SD* = 0.10, range = 0.05–0.47, higher values indicate non‐FSM students outperform FSM students to a greater degree).

## Results

We first conducted confirmatory factor analysis on the three self‐report measures, which suggested they had good construct validity. Full results are reported in the Supplementary Information. We then tested our hypotheses and accounted for the clustering of students within schools by conducting multilevel regressions in MPlus Version 8 (Múthen & Múthen, 1998). We used maximum likelihood estimation with robust standard errors, which uses all available data and estimates standard errors that are robust against violations of the assumptions of linear models. All the continuous variables were standardized, except the school‐level socioeconomic attainment gap variable, which was grand‐mean centred.

We first specified a model (Model 1) that regressed attainment 8 scores on the individual‐level variables FSM, female, EAL, and KS2 score. The results showed that all the predictor variables were associated significantly with attainment 8 scores. Confirming H1, students eligible for FSM had lower attainment 8 scores than students not eligible for FSM. In addition, females had higher attainment 8 scores than males, students for whom English was an additional language had higher scores than students for whom English was not an additional language, and KS2 score was associated positively and strongly with attainment 8 score (Table 1).

**Table 1:** Results from Model 1 with attainment 8 as the outcome

	Coefficient	Lower 95% CI	Higher 95% CI	*p*‐Value
Key Stage 2 score	0.656	0.619	0.692	<.001
Free School Meals eligibility	−0.354	−0.410	−0.297	<.001
Female	0.240	0.188	0.293	<.001
EAL	0.360	0.247	0.472	<.001
Residual variance	0.436	0.400	0.472	<.001

Note

All predictors are at the individual level of analysis. The model has seven free parameters.

EAL = English as an Additional Language.

Next, in Model 2, we added the individual‐level variables identity compatibility, academic effort, and academic self‐concept to the model as fixed effects and investigated whether there were indirect effects from the student characteristics (FSM, female, EAL) to attainment 8 via these psychological variables. Confirming Hypotheses 2–4, FSM negatively predicted identity compatibility, identity compatibility positively predicted attainment 8 scores, and there was a significant and negative indirect effect from FSM to attainment 8 via identity compatibility. Identity compatibility was significantly associated with attainment 8 over and above student characteristics and the other psychological variables, and was the only psychological variable to be associated significantly with FSM.

In addition, EAL positively predicted identity compatibility, EAL and female positively predicted academic effort, EAL positively predicted academic self‐concept, and female negatively predicted academic self‐concept. Academic effort and academic self‐concept also predicted attainment 8 scores. We also observed significant indirect effects from EAL to attainment 8 via identity compatibility and academic self‐concept, and from female to attainment 8 via academic effort and via academic self‐concept (Table 2).

**Table 2:** Level 1 direct and indirect effects from Model 2

Direct effects on…	Coefficient	Lower 95% CI	Higher 95% CI	*p*‐Value
…Attainment 8				
KS2 score	0.618	0.584	0.653	<.001
FSM	−0.333	−0.384	−0.281	<.001
Female	0.257	0.203	0.310	<.001
EAL	0.310	0.209	0.411	<.001
Identity compatibility	0.060	0.027	0.094	<.001
Academic effort	0.096	0.050	0.141	<.001
Academic self‐concept	0.118	0.081	0.156	<.001
…Identity compatibility				
FSM	−0.243	−0.343	−0.143	<.001
Female	−0.034	−0.103	0.036	.343
EAL	0.263	0.144	0.382	<.001
…Academic effort				
FSM	−0.062	−0.134	0.010	.089
Female	0.102	0.019	0.185	.017
EAL	0.138	0.007	0.270	.039
…Academic self‐concept				
FSM	−0.080	−0.177	0.018	.111
Female	−0.246	−0.335	−0.156	<.001
EAL	0.242	0.129	0.355	<.001
Residual variance Attainment 8	0.448	0.362	0.431	<.001
Indirect effects on attainment 8				
…via identity compatibility				
FSM	−0.015	−0.024	−0.005	.003
Female	−0.002	−0.006	0.002	.350
EAL	0.016	0.005	0.027	.005
…via academic effort				
FSM	−0.006	−0.014	0.002	.129
Female	0.010	0.001	0.018	.021
EAL	0.013	−0.002	0.029	.080
…via academic self‐concept				
FSM	−0.009	−0.022	0.003	.133
Female	−0.029	−0.041	−0.017	<.001
EAL	0.029	0.014	0.043	<.001

Note

All predictors are at the individual level of analysis. The model has 25 free parameters.

EAL = English as an Additional Language; FSM = Free School Meals eligibility; KS2 = Key Stage 2 (corresponding to age 11).

**Figure 2:**
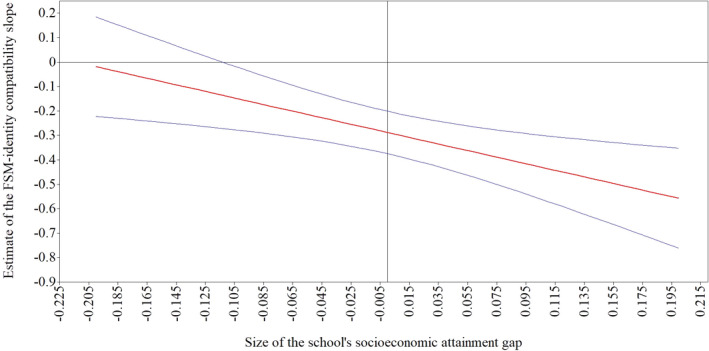
Graph showing how the unstandardized estimate of the FSM – identity compatibility slope varies across schools with different social class gaps. *Note* The solid straight line is the point estimate of the FSM‐identity compatibility slope, whereas the curved lines are the 95% confidence intervals.

Across the next few models, we allowed the FSM‐identity compatibility, FSM‐academic effort, FSM‐academic self‐concept, FSM‐attainment 8, and identity compatibility‐attainment 8 slopes to vary across schools. To maximize power, we specified one random slope per model. All the slopes varied significantly across schools.^2^ We then added SES attainment gap as a between‐level predictor and as a moderator of the random slope in a cross‐level interaction in each of the models. SES attainment gap did not predict Attainment 8, nor did it moderate the FSM‐academic effort slope, the FSM‐academic self‐concept slope, the FSM‐attainment 8 slope, or the identity compatibility‐attainment 8 slope. SES attainment gap did, however, moderate the FSM‐identity compatibility slope, confirming Hypothesis 5 (Model 3). As shown in Figure 2, the association between FSM and identity compatibility was negative in schools with a large SES attainment gap but was virtually zero in schools with a small SES attainment gap (Table 3).

**Table 3:** Results from Model 3 with attainment 8 as the outcome

Model 3				
	Coefficient	Lower 95% CI	Higher 95% CI	*p*‐Value
Outcome: Attainment 8				
Level 1 predictors				
KS2 score	0.618	0.584	0.653	<.001
FSM	−0.331	−0.381	−0.280	<.001
Female	0.256	0.203	0.310	<.001
EAL	0.311	0.210	0.412	<.001
Identity compatibility	0.062	0.030	0.095	<.001
Effort	0.095	0.049	0.141	<.001
Academic self‐concept	0.118	0.080	0.155	<.001
Level 2 predictors				
SES gap	−0.075	−0.807	0.656	.840
Cross‐level interaction				
SES gap * identity compatibility	−1.346	−2.269	−0.423	.004
Outcome: Identity compatibility				
Level 1 predictors				
FSM	−0.288	−0.375	−0.201	<.001
Female	−0.020	−0.089	0.049	.565
EAL	0.212	0.090	0.335	.001
Outcome: Effort				
Level 1 predictors				
FSM	−0.062	−0.133	0.010	.092
Female	0.102	0.018	0.185	.017
EAL	0.138	0.007	0.270	.039
Outcome: Academic self‐concept				
Level 1 predictors				
FSM	−0.079	−0.177	0.019	.112
Female	−0.246	−0.335	−0.156	<.001
EAL	0.242	0.129	0.355	<.001
Residuals				
Residual variance Attainment 8	0.397	0.362	0.431	<.001
Residual variance Random Slope	0.034	0.007	0.062	.014

Note

The model has 28 free parameters.

EAL = English as an Additional Language; FSM = Free School Meals eligibility; KS2 = Key Stage 2 (corresponding to age 11).

## General discussion

We report the first empirical evidence that the socioeconomic gap in academic attainment is partially explained by lower levels of identity compatibility among low SES students. Previous research has found that identity compatibility helps to explain the relationship between college students’ SES and the ranking of the university they plan to apply to (Nieuwenhuis et al., 2019), and between university students’ SES and their identification with university, wellbeing (Iyer et al., 2009; Jetten et al., 2008), and academic performance (Veldman et al., 2019). We extend this body of work by applying the concept of identity compatibility to school students. We found that perceptions of identity compatibility among 15–16‐year‐old school students in England positively predicted their performance in high‐stake national exams that were taken 8 months after the measurement of identity compatibility, and that it did so over and above students’ gender, English language status, academic effort, academic self‐concept, and prior exam results. It is noteworthy that the only psychological variable that was associated with FSM was identity compatibility, showing the importance of identity compatibility in explaining the socioeconomic attainment gap. Our results demonstrate that identity compatibility is an important social perception that predicts hard academic outcomes among school students and contributes to the socioeconomic attainment gap.

It is important to note, however, that we also found that the association between FSM and identity compatibility was moderated by school context. The results showed that FSM students reported lower levels of identity compatibility than their peers, but only when they attended schools that had relatively large socioeconomic attainment gaps in the previous academic year. In schools with small or non‐existent socioeconomic attainment gaps, there was no association between FSM and identity compatibility.

Interestingly, while the other associations in our model varied across schools, it was only the FSM‐identity compatibility slope that was moderated by the size of the socioeconomic attainment gap. These results provide support for the identities in context model of educational inequalities (Easterbrook & Hadden, 2021; Easterbrook et al., 2019), which attempts to describe the features of the local educational context – which include a group’s historical performance – that moderate the association between students’ group memberships and their experience of threat and perception of identity compatibility. The model stipulates that sociocultural factors in the local school context can change the meaning of students’ social identities and thus their subjective experience of school, with indirect effects on their performance via identity compatibility and threat (see also Manstead et al., 2020). It follows that only social psychological variables that incorporate some aspect of students’ social identities should be moderated by the sociocultural variables outlined by the model, as indeed we found.

Although this study was primarily testing theoretical predictions, it is worth considering the practical implications for interventions. Our results suggest that interventions that can reduce identity incompatibility – such as interventions that develop academic possible selves and/or raise the visibility of ingroup role models (Andriessen et al., 2006; Hernandez et al., 2017; Oyserman et al., 2006; Stout et al., 2011) – may reduce the socioeconomic attainment gap in English schools. However, these are likely to be effective only in schools that have historically large socioeconomic attainment gaps. To avoid wasting resources, interventionists should therefore gain a deep understanding of the local social context before they intervene (Easterbrook & Hadden, 2021).

As well as a group’s historical performance, the identities in context model stipulates that negative stereotypes, the visibility of academically successful role models, representation in positions and institutions associated with academic success, and a group’s cultural orientation towards education can all moderate the association between students’ group membership and their sense of threat and perception of identity compatibility (Easterbrook et al., 2019). These sociocultural factors should therefore help to explain any observed heterogeneity in the associations between students’ group memberships and a sense of threat and identity compatibility across contexts. Indeed, the model and this initial test of its predictions can be seen as efforts to incorporate and quantify the role of the local educational context in educational inequalities, and were in part inspired by the context sensitivity of ‘wise’ psychological intervention effects (Borman et al., 2018; Goroff, Lewis, Scheel, Scherer, & Tucker, 2018; Walton & Wilson, 2018) and the predicted heterogeneity revolution in behavioural science (Bryan, Tipton, & Yeager, 2021; Easterbrook, Harris, & Sherman, 2021). From this perspective, heterogeneity of effects is not a failure of psychological science, as the replication crisis often frames it, but an opportunity for developing theories and studies that account for contextual moderators of effects. The identities in context model and the analyses we report here are attempts to seize this opportunity.

Besides the findings related to our theoretical predictions, we also found evidence for a gender attainment gap, in which girls outperformed boys, which was partly accounted for by greater self‐reported effort among girls (Yeung, 2011). However, we also found a negative indirect effect of being a girl on attainment 8 scores via lower levels of academic self‐concept, contrary to previous research that has found that gender inequalities in subject specific academic self‐concepts (such as mathematics) tend to map onto gender inequalities in attainment (Sullivan, 2009). We also found that EAL students outperformed non‐EAL students in attainment 8 scores, in line with the latest national figures (Department for Education, 2020), and this was in part accounted for by higher levels of identity compatibility and academic self‐concept among EAL students.

### Strengths and limitations

There are several noteworthy strengths of the current research. We utilized a large sample of students across 29 schools and analysed the English Government’s preferred measure of academic attainment, attainment 8 scores. We also controlled for students’ gender, EAL, academic effort, academic self‐concept, and, crucially, prior academic performance within our models, allowing us to demonstrate that identity compatibility is uniquely associated with academic ability over and above these other variables. The survey measures were collected some 8 months before the exams that comprised our outcome measures, implying that the effects associated with students’ identity compatibility persist at least over the course of one full academic year.

There were also some limitations of our research designs that could be improved in future research. Our results are based on correlational data and so do not allow us to draw conclusions about causality. However, the fact that identity compatibility was measured before the exams that our outcome measures were based on – by 8 months – does confirm the directionality of the relationships, if not their causal nature. We also could not include ethnicity in our models, and so can make no conclusions about whether identity compatibility plays a role in ethnic attainment gaps. This is an important gap in our knowledge that should be addressed in future research.

### Conclusion

In summary, our results demonstrate the importance of social perceptions in explaining educational inequalities, and of understanding the role of the local educational context in determining which groups, if any, may be experiencing psychological barriers to their educational success. Students who are members of groups which have historically underperformed may be at a *psychological* disadvantage because of social cues in the local context that are perceived as indicating that their social identity is incompatible with academic success. Our work suggests that treating academic success as something *everyone* can achieve may help to make educational opportunities less unequal.

## Conflicts of interest

All authors declare no conflict of interest.

## Author contribution

**Matthew J. Easterbrook:** Conceptualization; Data curation; Formal analysis; Funding acquisition; Investigation; Methodology; Project administration; Supervision; Visualization; Writing – original draft. **Marlon Nieuwenhuis:** Conceptualization; Data curation; Funding acquisition; Investigation; Methodology; Project administration; Writing – review & editing. **Kerry J. Fox:** Data curation; Investigation; Project administration; Writing – review & editing. **Peter R. Harris:** Conceptualization; Funding acquisition; Project administration; Writing – review & editing. **Robin Banerjee:** Writing – review & editing.

## Supporting information


**Appendix S1**. Supplementary Information.

## Data Availability

The data that support the findings of this study are available on request from the corresponding author. The data are not publicly available due to privacy or ethical restrictions.

## Footnotes

^1^
The first of three interventions was implemented in English classes at least 1 week after the survey was completed, the second in December or January, and the final in April or May 2017. Teachers and students were blind to condition and the intervention was part of normal classroom activities, meaning students were unaware of their participation. Including condition as a predictor of Attainment 8 in the final model yielded almost identical results, with coefficient estimates varying from those produced by the model without condition by Δ*b* < 0.002. The estimate for condition was non‐significant (*b* = −0.016, *p* = .374).

^2^
The FSM‐identity compatibility slope varied significantly across schools (*estimate* = −0.28, 95% CIs [−0.37, −0.19], *p* < .001), as did the FSM‐academic effort slope (*estimate* = −0.08, 95% CIs [−0.14, −0.01], *p* = .034), the FSM‐academic self‐concept slope (*estimate* = −0.12, 95% CIs [−0.21, −0.02], *p* = .017), the FSM‐attainment 8 slope (*estimate* = −0.33, 95% CIs [−0.38, −0.27], *p* < .001), and the identity compatibility‐attainment 8 slope (*estimate* = −0.06, 95% CIs [−0.04, −0.09], *p* < .001).
