# Supplementary material for: ‘People like me don’t do well at school’: The roles of identity compatibility and school context in explaining the socioeconomic attainment gap
Source: Br J Educ Psychol. 2022 Mar 1;92(3):1178–95. doi: 10.1111/bjep.12494 (PMC9542118; doi:10.1111/bjep.12494)
Supplement: Supplementary file 1 — Appendix S1. Supplementary Information. [file BJEP-92-1178-s001.docx]

**Supplementary Online Materials**

**“People Like Me Don’t Do Well at School”: The Roles of Identity Compatibility and School Context in Explaining the Socioeconomic Attainment Gap**

**Contents:**

1. Sample Size Information
2. Table S1: Descriptive statistics for the analysed sample
3. Table S2: Descriptive statistics after listwise deletion
4. Table S3: Level 1 correlation matrix for the analysed sample.
5. Construct Validity
6. Adapted Measures
7. Mplus Syntax for Model 3

**Sample Size Information**

5,079 students took part in the intervention as part of the larger research study at the end of the academic year prior to the one in which we collected data. We had data for 4,629 students on Attainment 8 scores, Free School Meal (FSM) eligibility, gender, English as an Additional Language (EAL), and prior attainment. We report the characteristics of this sample in the main manuscript. However, 3,125 students completed the identity compatibility questions in the survey (68%), 3,231 competed the academic effort questions, and 3,222 completed the academic self-concept questions. The drop in sample size from randomisation to the survey was because two full schools and some classes within some of the other schools did not complete or return the surveys, a substantial proportion of students left the schools after randomisation but before the survey (i.e. during the summer break), and a few students were absent during data collection. Our analyses are based on the sample for which we have the primary outcome and predictor data (i.e. *n* = 4,629, *k* = 29).

Listwise deletion on all variables gives a sample size of *n* = 3,117, *k* = 27. The descriptive characteristics of this reduced sample are shown in Table S2.

**Descriptive Statistics for the Sample**

**Table S1**

*Descriptive statistics for the analysed sample*

|  | *n* | *k* | Mean | Variance | Standard Deviation | Skew | Kurtosis | Minimum | Maximum | Median | ICC1 | ICC2 |
| --- | --- | --- | --- | --- | --- | --- | --- | --- | --- | --- | --- | --- |
| *Individual level variables* |  |  |  |  |  |  |  |  |  |  |  |  |
| Attainment 8 | 4629 | 29 | 44.60 | 316.39 | 17.79 | -0.12 | -0.36 | 0.00 | 87.50 | 45.00 | 0.10 | 0.76 |
| Identity compatibility | 3125 | 27 | 4.37 | 1.44 | 1.20 | -0.23 | 0.29 | 1.00 | 7.00 | 4.33 | 0.03 | 0.43 |
| Effort | 3231 | 27 | 5.30 | 1.05 | 1.03 | -1.01 | 1.54 | 1.00 | 7.00 | 5.50 | 0.01 | 0.29 |
| Academic self-concept | 3222 | 27 | 4.58 | 1.50 | 1.22 | -0.45 | 0.01 | 1.00 | 7.00 | 4.68 | 0.02 | 0.37 |
| Free School Meal (FSM) eligibility | 4629 | 29 | 0.27 | 0.20 | 0.44 | 1.04 | -0.92 | 0.00 | 1.00 | 0.00 | 0.08 | 0.70 |
| Female | 4629 | 29 | 0.51 | 0.25 | 0.50 | -0.06 | -2.00 | 0.00 | 1.00 | 1.00 | 0.08 | 0.71 |
| EAL | 4629 | 29 | 0.08 | 0.07 | 0.27 | 3.08 | 7.50 | 0.00 | 1.00 | 0.00 | 0.22 | 0.89 |
| Prior performance | 4629 | 29 | 69.17 | 239.87 | 15.49 | -0.40 | -0.39 | 56.00 | 74.50 | 70.50 | 0.07 | 0.70 |
| *School-level variables* |  |  |  |  |  |  |  |  |  |  |  |  |
| Socioeconomic attainment gap |  | 29 | 0.00 | 0.01 | 0.09 | -0.04 | -0.37 | -0.20 | 0.22 | 0.00 | n/a | n/a |
|  |  |  |  |  |  |  |  |  |  |  |  |  |
|  |  |  |  |  |  |  |  |  |  |  |  |  |

**Table S2**

*Descriptive statistics after listwise deletion*

|  | *n* | *k* | Mean | Variance | Standard Deviation | Skew | Kurtosis | Minimum | Maximum | Median | ICC1 | ICC2 |
| --- | --- | --- | --- | --- | --- | --- | --- | --- | --- | --- | --- | --- |
| Attainment 8 | 3117 | 27 | 46.29 | 272.59 | 16.51 | -0.03 | -0.38 | 0.00 | 87.50 | 46.50 | 0.10 | 0.77 |
| Identity compatibility | 3117 | 27 | 4.37 | 1.44 | 1.20 | -0.24 | 0.29 | 1.00 | 7.00 | 4.33 | 0.02 | 0.41 |
| Effort | 3117 | 27 | 5.30 | 1.04 | 1.02 | -1.00 | 1.50 | 1.00 | 7.00 | 5.50 | 0.02 | 0.33 |
| Academic self-concept | 3117 | 27 | 4.59 | 1.50 | 1.23 | -0.43 | 0.01 | 1.00 | 7.00 | 4.67 | 0.02 | 0.41 |
| Free School Meal (FSM) eligibility | 3117 | 27 | 0.25 | 0.19 | 0.43 | 1.14 | 0.70 | 0.00 | 1.00 | 0.00 | 0.08 | 0.72 |
| Female | 3117 | 27 | 0.53 | 0.25 | 0.50 | -0.11 | 1.99 | 0.00 | 1.00 | 1.00 | 0.09 | 0.73 |
| EAL | 3117 | 27 | 0.08 | 0.07 | 0.27 | 3.16 | 7.96 | 0.00 | 1.00 | 0.00 | 0.25 | 0.91 |
| Prior performance | 3117 | 27 | 69.33 | 237.13 | 15.40 | -0.42 | -0.34 | 20.50 | 99.00 | 70.50 | 0.06 | 0.66 |
| Socioeconomic attainment gap |  | 27 | 0.00 | 0.01 | 0.09 | -0.07 | -0.67 | -0.20 | 0.22 | 0.00 | n/a | n/a |

**Table S3**

*Correlation matrix for the analysed sample.*

|  | Variable | 1 | 2 | 3 | 4 | 5 | 6 | 7 | 8 |
| --- | --- | --- | --- | --- | --- | --- | --- | --- | --- |
| 1 | Attainment 8 | - |  |  |  |  |  |  |  |
| 2 | Identity compatibility | 0.259 | - |  |  |  |  |  |  |
| 3 | Academic effort | 0.279 | 0.367 | - |  |  |  |  |  |
| 4 | Academic self-concept | 0.391 | 0.401 | 0.511 | - |  |  |  |  |
| 5 | Free school meal (FSM) eligibility | -0.272 | -0.111 | -0.034 | -0.036 | - |  |  |  |
| 6 | Female | 0.126 | -0.015 | 0.052 | -0.121 | 0.001 | - |  |  |
| 7 | English as an Additional Language (EAL) | 0.063 | 0.068 | 0.037 | 0.067 | 0.051 | -0.028 | - |  |
| 8 | Prior performance | 0.699 | 0.119 | 0.103 | 0.288 | -0.177 | 0.013 | -0.045 | - |
| 9 | Socioeconomic attainment gap (L2) | -0.027 | .001 | .001 | .001 | .001 | .001 | .001 | .001 |

**Construct Validity**

***Confirmatory Factor Analysis***

To investigate the construct validity of the three self-report measures—identity compatibility, academic effort, and academic self-construct—we specified a confirmatory factor analysis model in which the three constructs were specified as latent variables with their respective items as indicators. We included covariances between the latent factors, but not between any indicators. This model showed an excellent fit to the data with all standardised loadings > .47: χ^2^ (32) = 260.01, *p* < .001, CFI = .982, TLI = .974, RMSEA = .045 90% C.I. [.040, .050], SRMR = .027. This implies construct validity.

***Reliability***

Identity compatibility: McDonald’s Ω = .853, *p* < .001

Academic effort: McDonald’s Ω = .880, *p* < .001

Academic self-construct: McDonald’s Ω = .834, *p* < .001

***Inter-item Correlations***

Inter-item correlations between the identity compatibility items, and between the effort items, were all r >.90, and the academic self-concept items had inter-item correlations of r > .60.

**Adapted measures**

We slightly modified the four items from the original Student Approaches to Learning questionnaire by Marsh et al. (2006) to measure academic effort. The original wording, as reported in Marsh et al. (2006), was:

- When studying, I work as hard as possible.
- When studying, I keep working even if the material is difficult.
- When studying, I try to do my best to acquire the knowledge and skills taught.
- When studying, I put forth my best effort.

Our items had the preface “How do you feel about school work?”, and were

- I work as hard as possible
- I keep working even if the material is difficult
- I try my best to learn what is being taught
- I apply my best effort

**Mplus Syntax for Model 3:**

VARIABLES: [...]

missing are all(-999);

cluster is schoolid;

usevar

att8 prior fsm female eal idcomp effort asc

sesGap;

within are

fsm female eal01 idcomp effort asc prior;

between are

sesGap;

ANALYSIS: type = twolevel random;

OUTPUT: stdyx; cinterval;

DEFINE:

standardize

ks4_att8 idcomp effort asc prior;

center

sesGap (grandmean);

MODEL:

%within%

att8 on

prior fsm female eal01

idcomp effort asc;

a | idcomp on fsm;

idcomp on female;

idcomp on eal01;

effort on fsm;

effort on female;

effort on eal01;

asc on fsm;

asc on female;

asc on eal01;

%between%

att8 on sesGap;

a on sesGap;

*Note:* Prior = Key Stage 2 exam results; FSM = Free school meals; EAL = English as an additional language; idcomp = identity compatibility; asc = academic self-concept; att8 = attainment 8.
